# A novel *de novo* truncating variant in a Hungarian patient with CTNNB1 neurodevelopmental disorder

**DOI:** 10.1186/s12887-023-04509-w

**Published:** 2024-01-15

**Authors:** Nikoletta Nagy, Margit Pál, Dóra Nagy, Barbara Anna Bokor, Aliz Zimmermann, Balázs Gellén, András Salamon, László Sztriha, Péter Klivényi, Márta Széll

**Affiliations:** 1 Department of Medical Genetics, University of Szeged, Szeged, Hungary; 2 Functional Clinical Genetic Research Group of the HUN-REN and the University of Szeged, Szeged, Hungary; 3 Institute of Medical Genetics, Kepler University Hospital Med Campus IV, Johannes Kepler University Linz, Linz, Austria; 4 Department of Pediatrics, Szent-Györgyi Albert Medical Center, University of Szeged, Szeged, Hungary; 5 Department of Neurology, University of Szeged, Szeged, Hungary

**Keywords:** CTNNB1, Neurodevelopmental disorder, High-throughput, Truncating, Whole exome sequencing

## Abstract

**Purpose:**

We aimed to elucidate the underlying disease in a Hungarian family, with only one affected family member, a 16-year-old male Hungarian patient, who developed global developmental delay, cognitive impairment, behavioral problems, short stature, intermittent headaches, recurrent dizziness, strabismus, hypermetropia, complex movement disorder and partial pituitary dysfunction. After years of detailed clinical investigations and careful pediatric care, the exact diagnosis of the patient and the cause of the disease was still unknown.

**Methods:**

We aimed to perform whole exome sequencing (WES) in order to investigate whether the affected patient is suffering from a rare monogenic disease.

**Results:**

Using WES, we identified a novel, *de novo* frameshift variant (c.1902dupG, p.Ala636SerfsTer12) of the catenin beta-1 *(CTNNB1)* gene. Assessment of the novel *CTNNB1* variant suggested that it is a likely pathogenic one and raised the diagnosis of CTNNB1 neurodevelopmental disorder (OMIM 615,075).

**Conclusions:**

Our manuscript may contribute to the better understanding of the genetic background of the recently discovered CTNNB1 neurodevelopmental disorder and raise awareness among clinicians and geneticists. The affected Hungarian family demonstrates that based on the results of the clinical workup is difficult to establish the diagnosis and high-throughput genetic screening may help to solve these complex cases.

**Supplementary Information:**

The online version contains supplementary material available at 10.1186/s12887-023-04509-w.

## What is known

**•** CTNNB1 neurodevelopmental disorder is featured by two main symptoms: cognitive impairment and exudative vitreoretinopathy. Additional manifestations include truncal hypotonia, peripheral spasticity, dystonia, behavioral problems, microcephaly, refractive errors, strabismus, intrauterine growth restriction, feeding difficulties, and scoliosis.

**•** CTNNB1 neurodevelopmental disorder develops as a consequence of *de novo* germline loss-of-function pathogenic variants of the catenin beta-1 *(CTNNB1)* gene. The CTNNB1 protein is a component of adherens junctions and contributes to the establishment and maintenance of epithelial layers and adhesion between cells. CTNNB1 is also involved in the WNT signaling pathway regulating several biological processes including cell proliferation or cell fate determination.

**What is new**

**•** The c.1902dupG, p.Ala636SerfsTer12 is a novel heterozygous frameshift variant in the 12th exon of the *CTNNB1* gene. Based on the ACMG variant classification guideline, this variant could be classified as a likely pathogenic one, since null variants (frameshift) in gene *CTNNB1* are predicted to cause loss-of-function, which is a known mechanism of the disease.

• The c.1902dupG, p.Ala636SerfsTer12 variant affects the 10th amino acid of the last 40 amino acid–long ARM domain of the encoded protein, and after 12 amino acids it results in the formation of a premature termination codon, which may either cause a nonsense-mediated RNA decay or a severely mutated protein with a largely truncated ARM domain and a missing 3’ end of the protein. ARM domains may mediate the interaction of CTNNB1 with its ligands; therefore, we hypothesized that the identified novel variant has a severe loss-of-function impact on protein functions.

**Supplementary Information:**

The online version contains supplementary material available at 10.1186/s12887-023-04509-w.

## Introduction

CTNNB1 neurodevelopmental disorder with spastic diplegia and visual defects (OMIM 615,075, also known as NEDSDV or CTNNB1-NDD) is hallmarked by two main symptoms: cognitive impairment and exudative vitreoretinopathy. Additional manifestations of this disease include truncal hypotonia, peripheral spasticity, dystonia, behavioral problems, microcephaly, refractive errors, and strabismus. Less frequent symptoms are intrauterine growth restriction, feeding difficulties, and scoliosis [1, 2]. CTNNB1 neurodevelopmental disorder shows autosomal dominant inheritance. It primarily develops as a consequence of de novo germline loss-of-function pathogenic variants of the catenin beta-1 *(CTNNB1)* gene [3]. The majority of disease-causing variants in the *CTNNB1* gene are truncations caused by frameshifts and nonsense variants [4].

Disease-causing variants in *CTNNB1* may lead to the development of exudative vitreoretinopathy type 7, of which the clinical symptoms do not include any cognitive impairment or neurological abnormalities [5]. Somatic variants of the *CTNNB1* gene are linked to various tumors, such as colorectal cancer, hepatocellular carcinoma, medulloblastoma, ovarian cancer, and pilomatricoma [6, 7].

*CTNNB1* encodes beta catenin, a member of the evolutionary conserved armadillo repeat proteins [8]. The CTNNB1 protein is a component of adherens junctions and contributes to the establishment and maintenance of epithelial layers and adhesion between cells. CTNNB1 is also involved in the WNT signaling pathway, which regulates various biological processes, including cell proliferation or cell fate determination [9].

Here, we present the case of a Hungarian patient affected by CTNNB1 neurodevelopmental disorder with a novel likely pathogenic frameshift variant p.Ala636SerfsTer12 in the *CTNNB1* gene. This likely pathogenic variant was not present in the clinically unaffected parents of the patient suggesting its *de novo* nature. This result increases the known *CTNNB1* variant spectrum associated with the CTNNB1 neurodevelopmental disorder.

## Materials and methods

### Patient

**Fig. 1:**
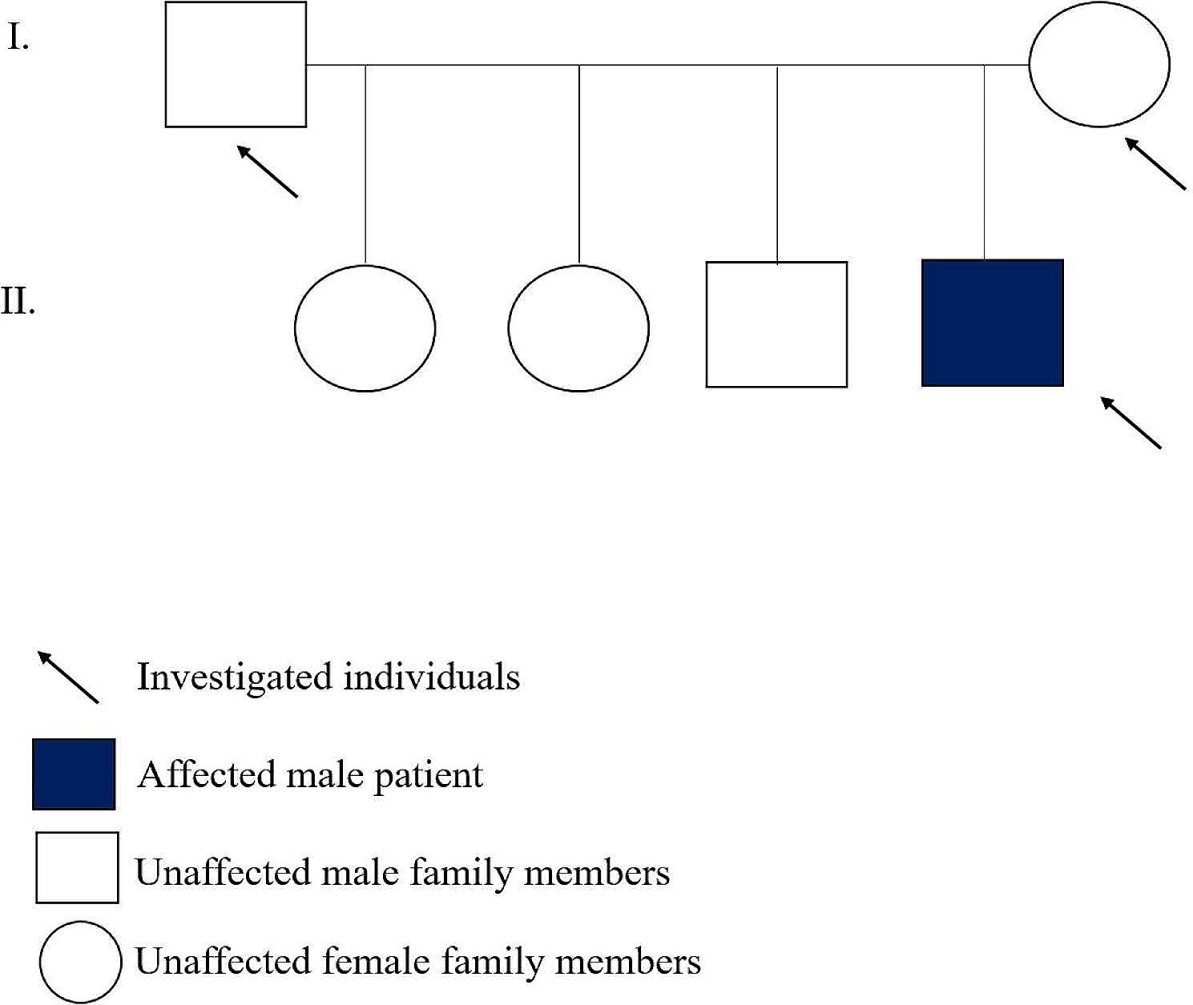
**Pedigree.** The Hungarian patient (IV/8) suffering from CTNNB1 neurodevelopmental disorder is the only affected member in his family

This study involved a 16-year-old male Hungarian patient who was born at 35 weeks gestation following an uncomplicated pregnancy. At birth, the head circumference was 31 cm, the birth weight was 2560 g and the birth length was 45 cm. During early childhood, the patient developed axial hypotonia and increased tone in all four limbs. Global developmental delay was observed. Cognitive impairment was detected with a borderline IQ. Behavioral problems were observed, which included aggressiveness and fits of anger. The patient was of short stature as his height was lower than the 3rd percentile. Intermittent headaches and recurrent dizziness were reported. Skull MRI of the 9-year-old patient demonstrated chronic sinusitis sphenoidalis, no other abnormality was present. Upon ophthalmologic examination, strabismus and hypermetropia were evident. During neurological examination, symptoms of complex movement disorder were detected with spasticity, slight ataxia, intermittent dystonia, stereotypes. Video demonstrates the complex movement disorder of the patient (Additional Video 1). When the video was recorded, the head circumference was 54 cm, body weight was 49.2 kg and body length was 162.6 cm (February 1, 2023). Because of partial pituitary dysfunction, growth hormone was administered at the age of 10. The patient responded well to the treatment. The patient is the only known affected family member. He has three clinically unaffected siblings, two sisters, and one brother (Fig. 1).

Written informed consent was obtained from the parents of the patient and genetic studies were conducted according to a protocol approved by the Hungarian National Public Health Center, in adherence with the Helsinki guidelines. The patient and his parents underwent pre- and post-test genetic counseling at the Department of Medical Genetics, University of Szeged (Szeged, Hungary).

### DNA extraction

Genomic DNA was extracted from venous blood mixed in the presence of the anticoagulant EDTA using the DNeasy® Blood & Tissue Kit (QIAGEN, Germany) as described in the manufacturer’s instructions. For quantification, a Qubit Fluorometric Quantification instrument was used according to the manufacturer’s instructions.

### Whole exome sequencing

The patient’s genotype was determined using next-generation sequencing (NGS). Library preparation was carried out using the SureSelectQXT Reagent Kit (Agilent Technologies, Santa Clara, CA). Pooled libraries were sequenced on an Illumina NextSeq 550 NGS platform using the 300-cycles Mid Output Kit v2.5 (Illumina, Inc., San Diego, CA, USA). Adapter-trimmed and Q30-filtered paired-end reads were aligned to the hg19 human reference genome using Burrows–Wheeler Aligner (BWA). Duplicates were marked using the Picard software package. The Genome Analysis Toolkit (GATK) was used for variant calling (BaseSpace BWA Enrichment Workflow v2.1.1. with BWA 0.7.7-isis-1.0.0, Picard: 1.79 and GATK v1.6-23-gf0210b3).

The mean on-target coverage achieved from sequencing was 71× per base and the average percentage of targets covered was greater or equal to 30× of 96% and 90%, respectively. Variants passed by the GATK filter were used for downstream analysis and annotated using the ANNOVAR software tool (version 2017 July 17) [10]. SNP testing was performed as follows: high-quality sequences were aligned with the human reference genome (GRCh37/hg19) to identify sequence variants. The detected variations were analyzed and annotated. Variants were filtered according to read depth, allele frequency, and prevalence in the genomic variant databases, asExAc (v.0.3) and Kaviar. Variant prioritization tools (PolyPhen2, SIFT, LRT, Mutation Taster, Mutation Assessor) were used to predict the functional impact. For variant filtering and interpretation, VarSome and Franklin bioinformatic platforms [https://franklin.genoox.com] were used according to the guidelines of the American College of Medical Genetics and Genomics [11, 12].

**Fig. 2:**
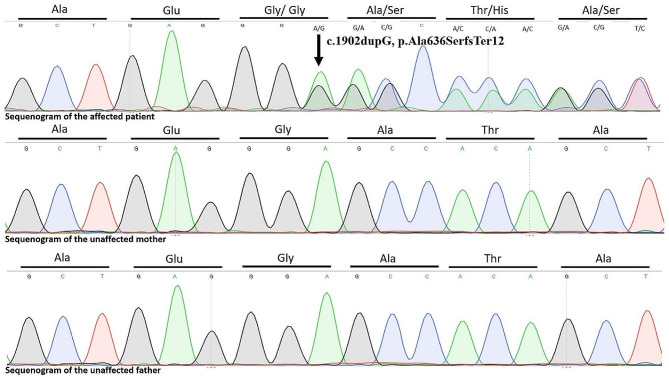
Sequenograms of the affected patient and the unaffected parents

The identified candidate variant was confirmed by bidirectional capillary sequencing in DNA from the patient and parents (Fig. 2).

## Results

**Fig. 3:**
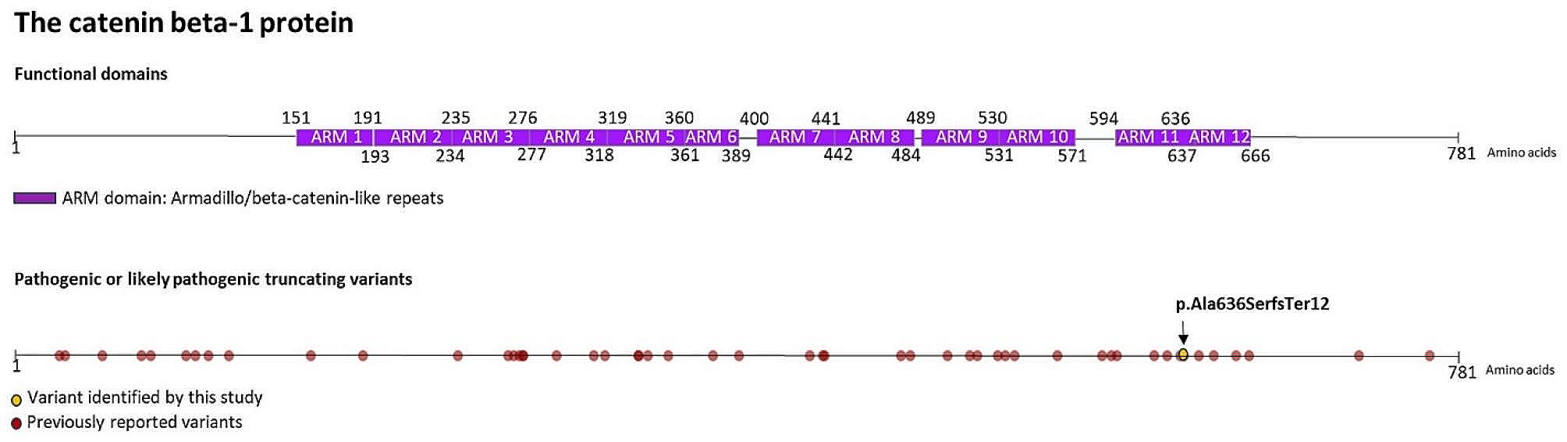
Location of the variant (c.1902dupG, p.E634fs) of the CTNNB1 protein

Whole exome sequencing identified a novel (c.1902dupG, p.Ala636SerfsTer12) heterozygous frameshift variant in the 12th exon of the *CTNNB1* gene 3p22.1, NM_001904.4 (Fig. 2). This variant was not detected in the parents of the patient, thus it is considered a *de novo* variant (PM6). Based on the ACMG variant classification guideline, this variant could be classified as a likely pathogenic one, since null variants (frameshift) in gene *CTNNB1* are predicted to cause loss-of-function, which is a known mechanism of the disease. The affected exon contains 11 other pathogenic and likely pathogenic variants and the truncated region contains 32 other pathogenic and likely pathogenic ones (PVS1) and it was not present in gnomAD population databases (PM2) (Additional Table 1). The detected novel frameshift variant affects the last armadillo/beta-catenin-like repeats domain (ARM domain) of the encoded protein (Fig. 3) (UniProt Tools, https://www.uniprot.org/uniprotkb/P35222/variant-viewer). The identified frameshift variant results in the formation of a premature termination codon, 12 amino acids after the variant. It affects the 10th amino acid of the last 40 amino acid–long ARM domain, and after 12 amino acids it results in the formation of a premature termination codon, which may either cause a nonsense-mediated RNA decay or a severely mutated protein with a largely truncated ARM domain and a missing 3’ end of the protein. ARM domains in general are composed of tandem repeats that form a superhelix of helices. They may mediate the interaction of CTNNB1 with its ligands; therefore, we hypothesized that the identified novel variant has a severe loss-of-function impact on protein functions. The region of the variant on the CTNNB1 protein exhibits high evolutionary conservation (Additional Fig. 1) (Aminode, http://www.aminode.org/search). *In silico* functional predictions using MT, DANN, BayesDel, SpliceAI, GERP, GenoCanyon, fitCons, and CADD also suggest that the newly identified frameshift variant has severe consequences and further supports its putative disease-causing role in the development of the observed CTNNB1 neurodevelopmental disorder (Additional Table 1).

## Discussion

We describe a Hungarian patient affected by a CTNNB1 neurodevelopmental disorder, which developed as a consequence of a newly identified, *de novo* frameshift variant in the *CTNNB1* gene (c.1902dupG, p.Ala636SerfsTer12). The patient is heterozygous for this variant. Both the clinical symptoms of the affected patient and the identified *de novo* heterozygous truncating variant on the *CTNNB1* gene are consistent with the typical symptoms of this disease and the most common genetic scenario involved in disease development [4]. The truncating variants including frameshift and nonsense ones, such as the newly identified p.Ala636SerfsTer12 variant, are the most common types of disease-causing variants of the gene. This is probably the result of nonsense-mediated RNA decay or the absence of one or more ARM domains in the encoded protein. The ARM domain is an approximate 40 amino acid–long tandemly repeated sequence motif, which mediates the interaction of the CTNNB1 protein with its ligands. Disruption of the interactions of the CTNNB1 protein with its ligands may lead to impaired synaptic plasticity, neuronal network connectivity, brain malformations, and consequently, to the development of the CTNNB1 neurodevelopmental disorder [13]. Concerning the less frequent symptoms of the CTNNB1 neurodevelopmental disorder related phenotype, the reported Hungarian patient has partial pituitary dysfunction and as a consequence he underwent growth hormone administration. Here, we emphasize that however it is less frequent manifestation, but still pathogenic variants of the *CTNNB1* gene can lead to the development of pituitary dysfunction.

Variants and genotype-phenotype correlations of 404 patients with CTNNB1 neurodevelopmental disorder suggest that the most common clinical feature in patients with pathogenic or likely pathogenic variants of the CTNNB1 gene is a mild-to-profound cognitive impairment. Exudative vitreoretinopathy, truncal hypotonia, peripheral spasticity, dystonia, behavior problems, microcephaly, refractive errors and strabismus are also frequently present. Less common clinical symptoms include intrauterine growth restriction, feeding difficulties, and scoliosis [4]. The above list of symptoms well-reflects the clinical heterogeneity of the disease, thus genetic testing might contribute to the establishment of the clinical diagnosis.

However, there is still no causative treatment for this severe condition, clinical and preclinical studies have identified several drugs targeting the WNT signaling pathway such as lithium, SB216763, sulindac, and PPARγ agonist, which may have therapeutic effects for NDDs [14]. The patients with CTNNB1 neurodevelopmental disorder are usually under supportive care by a multidisciplinary team. Therefore, genetic screening has a significant impact on the affected families by facilitating family planning and the birth of healthy children. However, most of the cases are de novo and the disease is rarely inherited from an affected parent [1, 2]. Germline mosaicism was reported in one family with two affected offspring and healthy parents [15]. If the *CTNNB1* pathogenic variant found in the proband cannot be detected in leukocyte DNA of either parent, the recurrence risk to siblings is estimated to be 1% because of the possibility of parental germline mosaicism [15]. Prenatal and/or preimplantation genetic testing should be available for affected families.

In this study, we identified a novel variant in the clinically and genetically homogenous CTNNB1 neurodevelopmental disorder. Hopefully, in the near future, these genetic discoveries will completely define the genetic background of the CTNNB1 neurodevelopmental disorder and provide a solid basis for studies developing novel therapeutic modalities for patients.

### Electronic supplementary material

Below is the link to the electronic supplementary material.

Supplementary Material 1

Supplementary Material 2

Supplementary Material 3

## Data Availability

The Department of Medical Genetics, University of Szeged, Hungary is registered in ClinVar and available at the following open access link: https://www.ncbi.nlm.nih.gov/clinvar/submitters/505686/. The identified likely pathogenic variant reported in this study and the associated phenotype are registered in ClinVar (SUB13804099), and available at the following open access link: VCV002579643.1 - ClinVar - NCBI (nih.gov).

## Abbreviations

ARM: Armadillo/beta-catenin-like repeats domain
CTNNB1: Catenin beta-1
NDD: Neurodevelopmental disorder
NEDSDV: CTNNB1 neurodevelopmental disorder
NGS: Next generation sequencing
PPARγ: Peroxisome proliferator-activated receptor gamma
