# Supplementary figures and images for: A novel de novo truncating variant in a Hungarian patient with CTNNB1 neurodevelopmental disorder
Source: BMC Pediatr. 2024 Jan 15;24:47. doi: 10.1186/s12887-023-04509-w (PMC10789033; doi:10.1186/s12887-023-04509-w)

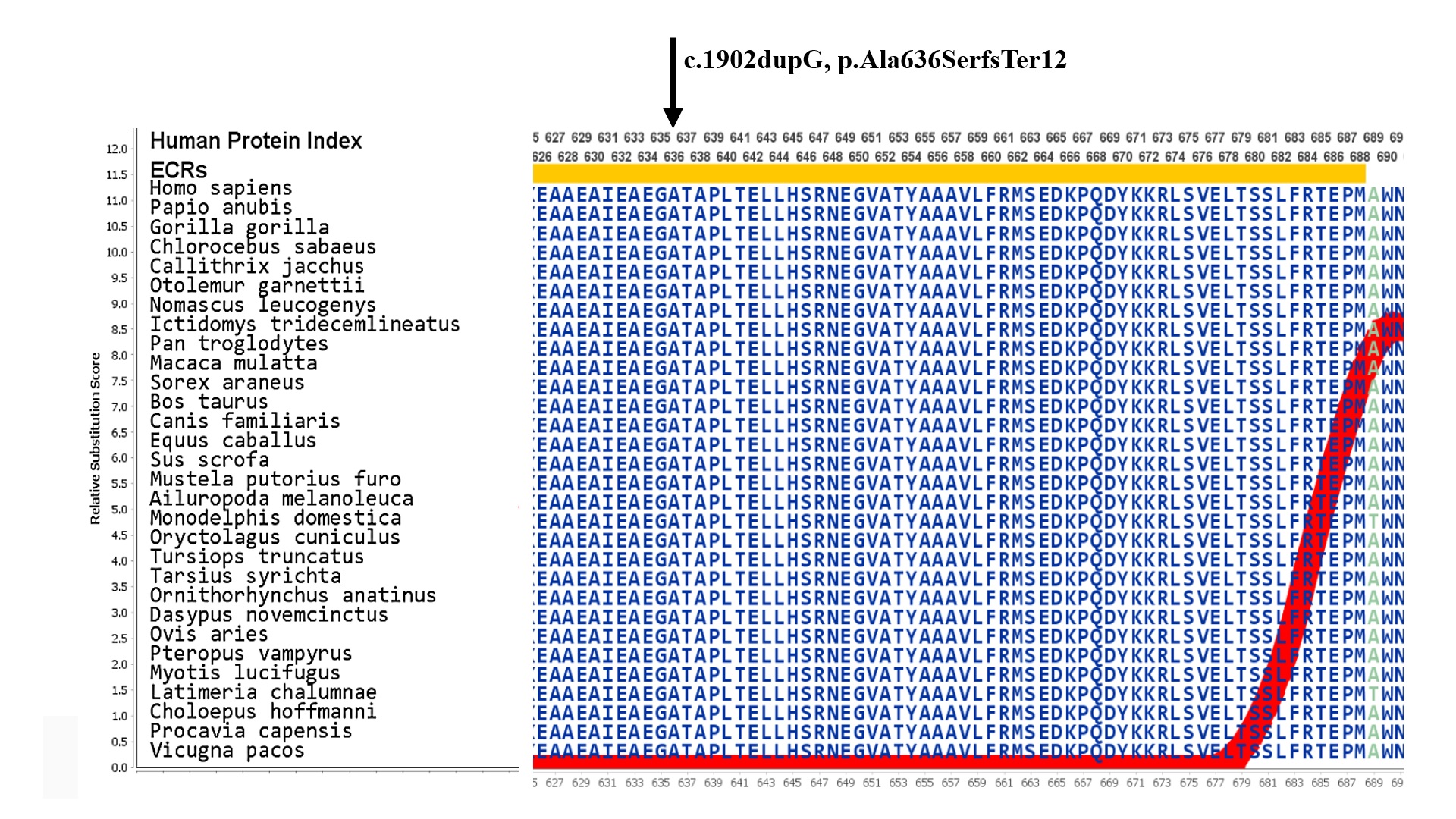

Supplement: Supplementary file 1 — Supplementary Material 1 [file 12887_2023_4509_MOESM1_ESM.jpg]
