# Supplementary material for: A novel de novo truncating variant in a Hungarian patient with CTNNB1 neurodevelopmental disorder
Source: BMC Pediatr. 2024 Jan 15;24:47. doi: 10.1186/s12887-023-04509-w (PMC10789033; doi:10.1186/s12887-023-04509-w)
Supplement: Supplementary file 3 — Supplementary Material 3 [file 12887_2023_4509_MOESM3_ESM.pdf]

Additional Table 1.

	***CTNNB1*:c.1902dupG p.E634fs**
**ACMG Classification**	Likely pathogenic (LP)
**Effect on Protein**	Pathogenic Very Strong (PVS1)
**Population data**	Pathogenic Moderate (PM2)
**gnomAD (Exome, Genome, Aggregated), TOPMed Bravo, GME Variome, Iranome, ExAC, 1000 Genomes, ESP 6500, 4.7KJPN, GenomeAsia, Mexican DB, India DB, Turkish Variome**	No Observation for this variant
**Revel, EVE, Varity, MUTAssessor, SIFT, Polyphen2, MT, FATHMM, DANN, MetaLR, PrimateAI, BayesDel, CardioBoost ARM, CardioBoost CM, dbscSNV Ada, dbscSNV RF, GERP, GenoCanyon, fitCons, Mitochondrial, MitoTip, APOGEE**	N/A
